# Five microRNAs in serum as potential biomarkers for prostate cancer risk assessment and therapeutic intervention

**DOI:** 10.1007/s11255-018-2009-4

**Published:** 2018-10-15

**Authors:** Xiaogang Guo, Tao Han, Pingping Hu, Xiaojun Guo, Changming Zhu, Youbao Wang, Shaoyan Chang

**Affiliations:** 1 Urology Department of Urology, Haici Medical Group of Qingdao, Qingdao, Shandong China; 2 Department of Cardiology, Jimo People’s Hospital, Qingdao, Shandong China; 3 Beijing Municipal Key Laboratory of Child Development and Nutriomics, Capital Institute of Pediatrics, Beijing, China

**Keywords:** Prostate cancer, MiR-1825, MiR-484, MiR-205, MiR-141, Let-7b

## Abstract

**Background:**

Prostate cancer (PCa) is a common malignant human tumor and one of the main causes of cancer-related deaths in men. At present, prostate-specific antigen levels are widely used to diagnose PCa in the clinic, but they are not sufficient for an accurate early diagnosis or prognosis.

**Methods:**

To identify potential molecular markers for PCa, we used real-time PCR to measure the expression levels of various microRNAs, including miR-1825, miR-484, miR-205, miR-141, and let-7b, in the serum of 72 PCa patients and 34 healthy controls.

**Results:**

miR-1825, miR-484, miR-205, miR-141, and let-7b were shown to be highly specific for PCa, suggesting that they could be used as PCa tumor screening biomarkers. miR-205 may also be used as a biomarker for indicating bone metastasis in PCa patients, miR-1825 levels may help indicate tumor–node–metastasis classification, the evaluation of treatment effects, and determining prognosis, while let-7b levels may indicate potential tumor malignancy and the hormone resistance status and could be used as a basis to adjust individual treatments for the high-risk, early diagnosis of refractory PCa.

**Conclusion:**

This study identified possible PCa tumor markers to more accurately predict the occurrence, progression, and prognosis of PCa, and which could be used in the development of tumor drug therapy.

**Electronic supplementary material:**

The online version of this article (10.1007/s11255-018-2009-4) contains supplementary material, which is available to authorized users.

## Introduction

Prostate cancer (PCa) is the most common malignant tumor in humans and one of the main causes of male cancer-related deaths [1]. Several tumor markers for PCa have already been identified, including prostate-specific antigen (PSA); however, these can be affected by confounding factors such as benign prostatic hyperplasia (BPH), prostate gland inflammation, and prostate massage [2].

Micro (mi) RNAs are non-coding, single-stranded RNA molecules approximately 22 nucleotides in length that are involved in regulating many biological processes [3]. They both suppress and activate the expression of genes by directly binding to target mRNAs for the degradation or inhibition of protein translation. Novel tumor markers are required for PCa to determine its development and progression, and miRNAs are a promising target for tumor drug therapy [4].

Whole genome expression profiling was previously used to identify possible roles for miRNAs 1234, 1238, 1913, 489-5p, 1825, 484, and 483-5p in the development, progression, or suppression of PCa [5]. Additionally, miR-205 is mainly expressed on the basal cells of prostate tissue, and inhibits the targets of zinc finger E-box-binding proteins, PKCε and HMGB3 to inhibit tumor proliferation and apoptosis and cancer cell aggressiveness [6, 7]. The let-7 family may also play an important role in the recurrence and metastasis of PCa by regulating tumor stem cells [8]. Let-7b was shown to inhibit the expression of enhancer of zeste homolog (EZH)2 and the growth of PCa cells, and low let-7b expression may be associated with PCa recurrence and tumor hormone resistance [9].

miR-141 is located on chromosome 12 and its pre-miRNA precursor produces two mature miRNAs, miR-141-3p and miR-141-5p, in the cytoplasm [10]. miRDB database analysis showed that the androgen receptor (AR) was a possible target gene of miR-141-3p, which was effective in targeted AR treatment of PCa [11, 12]. These studies provide a theoretical basis for the application of miRNAs in PCa, and suggest that they could be used as molecular diagnostic markers. However, many studies are limited to cellular investigations or the analysis of limited clinical samples.

In this study, we used real-time PCR to detect the expression of miRNAs, including miR-1825, miR-484, miR-205, miR-141, and let-7b, in 72 patients with PCa and 34 healthy controls. Several stratified analyses were carried out to study the expression of these miRNAs, with the aim of identifying potential molecular markers for the diagnosis and drug therapy assessment of PCa.

## Materials and methods

### Sample collection

We collected serum samples from 72 patients diagnosed with PCa by biopsy or surgical specimen pathology in our hospital from July 2015 to September 2016. Thirty-four healthy individuals were enrolled in the control group. They do not have prostatic hyperplasia confirmed by magnetic resonance and ultrasound and prostatitis examined from their prostatic fluid. Patients with a history or current diagnosis of tumors, diabetes, hypertension, urinary tract diseases such, or related immune or infectious diseases were excluded. Blood samples were collected from all participants in a vacuum tube of anticoagulation, then centrifuged at 3000×*g* for 10 min. The separated plasma was aliquoted and stored at − 80 °C. The study was approved by the ethics committee of the local institution and performed in accordance with the principles of the Declaration of Helsinki. Subject information is shown in Table 1.

**Table 1:** Characteristic of PCa and control

Characteristic	PCa	Control
N	72	34
Age	76.46	74.62
PSA treatments		
< 4 ng/ml	22	
> 4 ng/ml	50	
Gleason score		
6–7	29	
8–10	43	
TNM status		
T1–T2	39	
T3–T4	33	
Bone metastases		
Yes	34	
No	38	
Hormone dependency		
Dependence	23	
Resistance	25	

### miRNA extraction and reverse transcription

miRNA was extracted from serum samples using the Serum MicroRNA Extraction and Purification Kit (Shanghai Novland, Shanghai, China) and stored at − 80 °C. Reverse transcription was carried out at 16 °C for 30 min, 42 °C for 30 min, then 85 °C for 10 min. The reverse transcription system is shown in Table S1.

Real-time quantitative PCR was performed using primers shown in Table S2 at 95 °C for 3 min, then 40 cycles of 95 °C for 12 s, and 60 °C for 40 s. Real-time fluorescence quantitative response system is shown in Table S3. Fold changes were calculated using the 2^−ΔΔCT^ method [13].

### Statistical analysis

The experimental results were processed by SPSS version 17.0 software (McGraw-Hill Inc, New York, NY). Data are shown as means ± standard deviation, and were accurate to 0.01. Two independent groups were compared with *t* or rank sum tests. *p* < 0.05 was used to indicate statistical significance.

## Results

### miRNA levels in PCa patients and healthy control

**Fig. 1:**
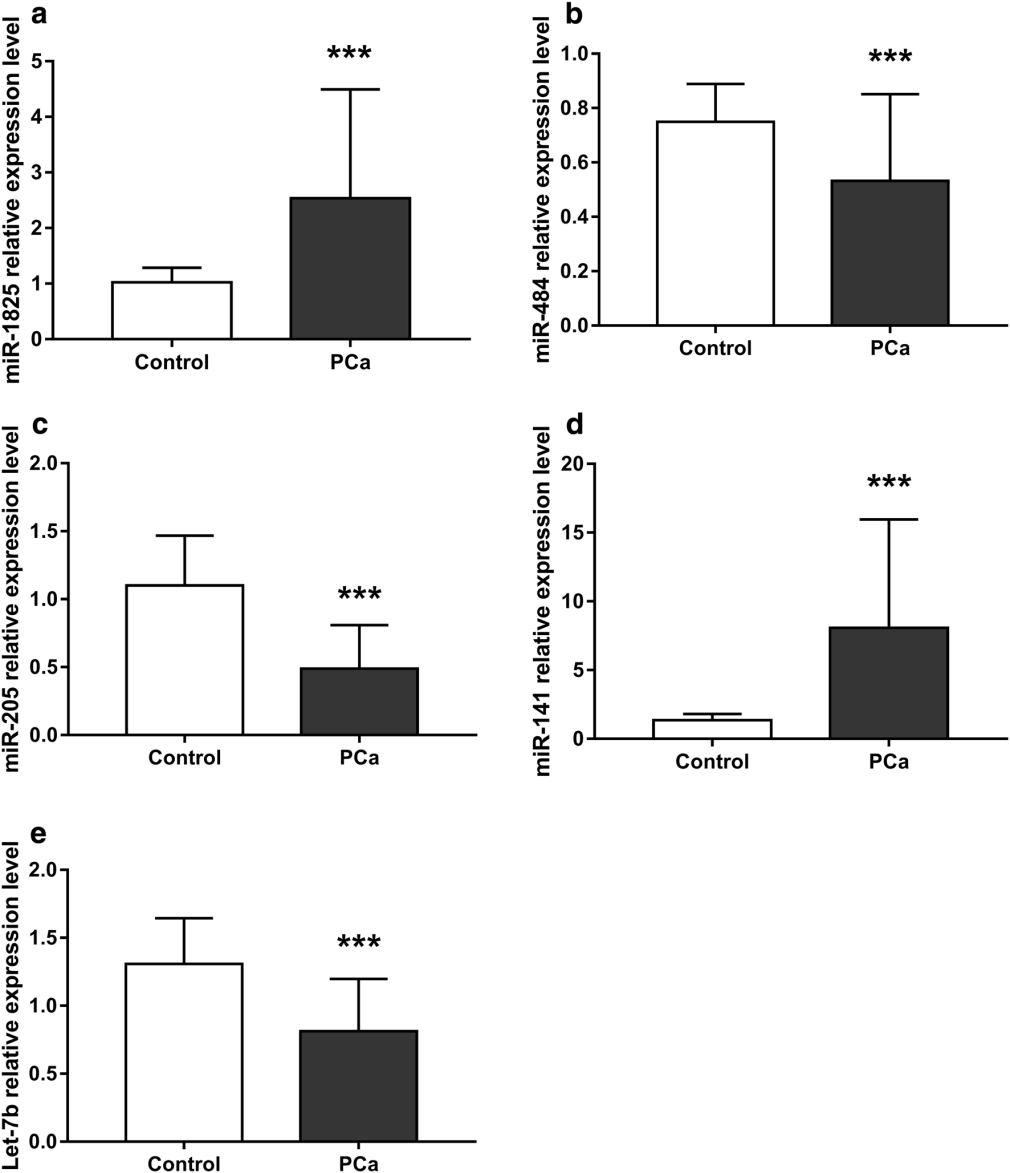
Relative miRNA expression levels between prostate cancer and control groups. **a** miR-1825 expression was significantly higher in PCa patients than controls. **b** miR-484 expression was significantly lower in PCa patients than controls. **c** miR-205 expression was significantly lower in PCa patients than in controls. **d** miR-141 expression was significantly higher in PCa patients than controls. **e** Let-7b expression was significantly lower in PCa patients than controls. PCa, prostate cancer group; control, healthy group. Results are shown as means ± SD. *p < 0.05, **p < 0.01, ***p < 0.001

We evaluated the levels of miR-1825, miR-484, miR-205, miR-141, and let-7b in the 72 PCa patients and 34 healthy controls, and found significant differences between the two groups (Table S4). In the PCa group, miR-484, miR-205, and let-7b levels were significantly lower than in the control group (Fig. 1b, c, e), while miR-1825 and miR-141 were significantly higher in the control group than the PCa group (Fig. 1a, d).

### miRNA levels in healthy controls and PCa patients with PSA < 4 ng/ml after PCa treatment

**Fig. 2:**
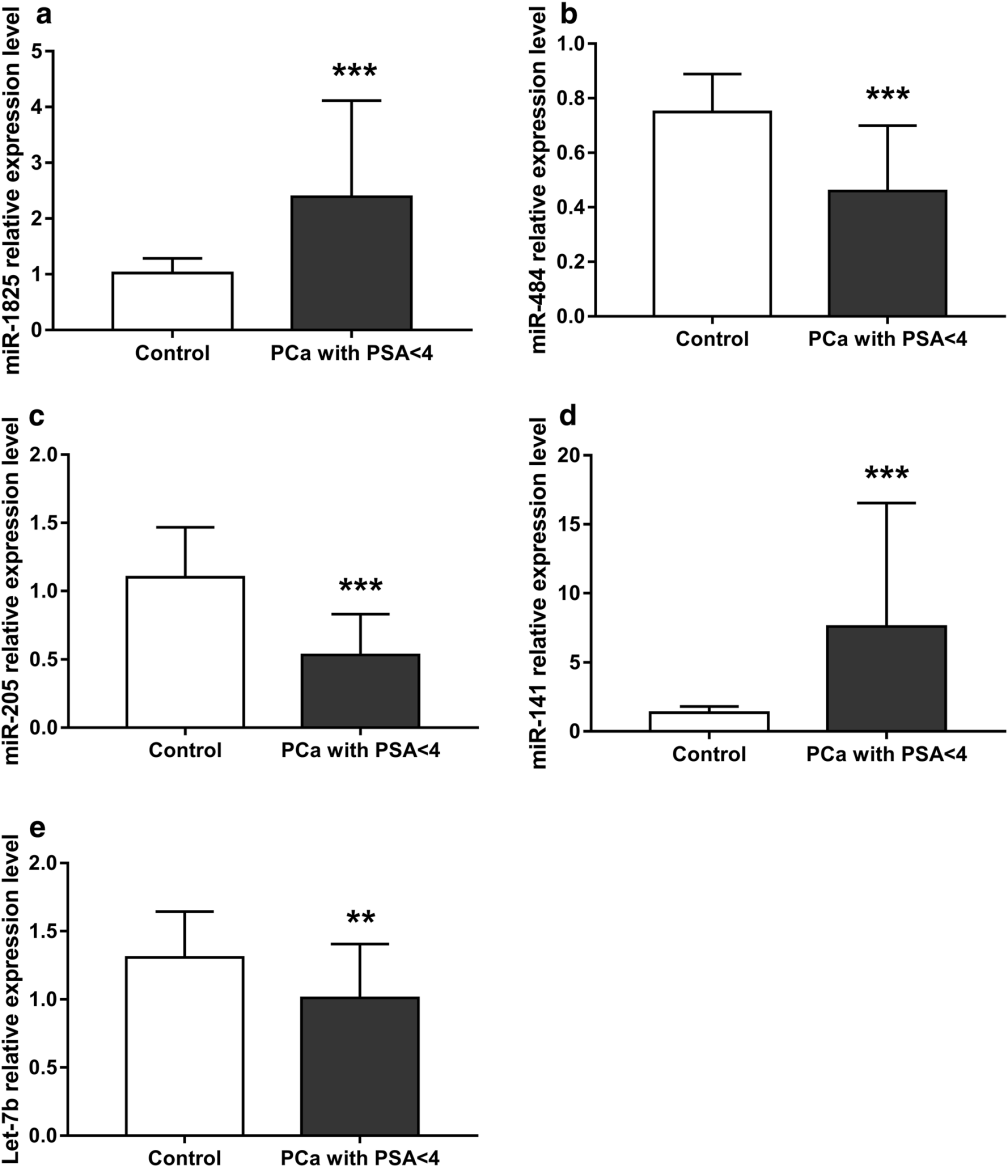
Relative miRNA expression levels between PCa patients with PSA (< 4 ng/ml) after treatment and control groups. **a** miR-1825 expression was significantly higher in PCa patients with PSA < 4 ng/ml than controls. **b** miR-484 expression was significantly lower in PCa patients with PSA < 4 ng/ml than controls. **c** miR-205 expression was significantly lower in PCa patients with PSA < 4 ng/ml than in control; **d** miR-141 was significantly higher in PCa with PSA < 4 than controls. **e** Let-7b expression was significantly lower in PCa patients with PSA < 4 ng/ml than controls. PCa with PSA < 4, prostate cancer patients with PSA < 4 ng/ml after treatment; control, healthy group. Results are shown as means ± SD. *p < 0.05, **p < 0.01, ***p < 0.001

We next examined miRNA levels in the healthy control group and 22 PCa patients whose PSA levels decreased to < 4 ng/ml after PCa treatment which included endocrine treatment, surgery, radiotherapy, or combined therapy for more than 1 month. We found a significant difference in the expression of all miRNAs examined between PCa patients who had undergone treatment and healthy controls (Fig. 2 and Table S5).

### miRNA levels before and after treatment in the same PCa patients

**Fig. 3:**
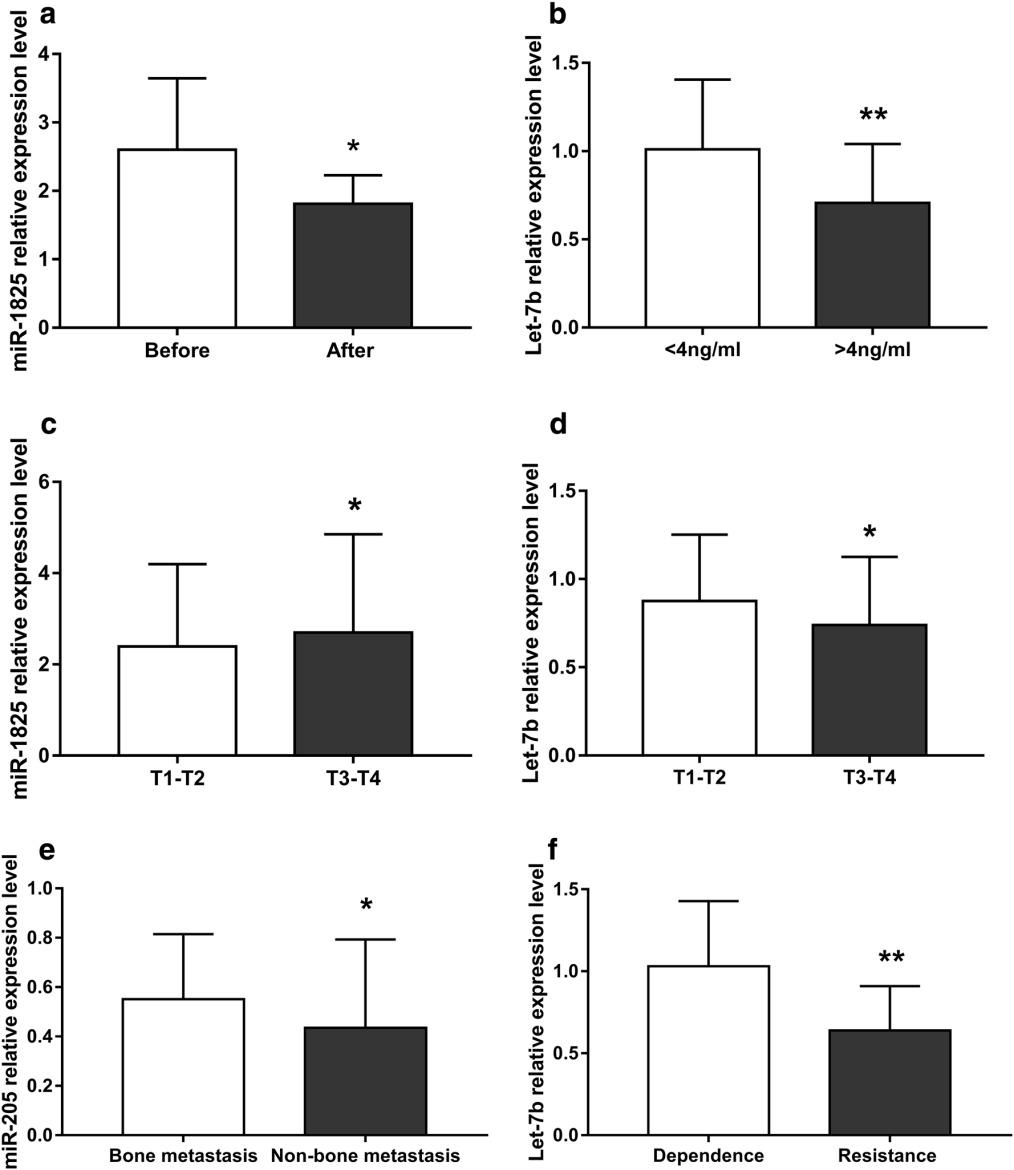
Relative miRNA expression in multiple stratified analysis. **a** miR-1825 expression was significantly higher before treatment than after. **b** Let-7b expression was significantly higher in patients with PSA < 4 ng/ml than those with PSA > 4 ng/ml. **c** miR-1825 expression was significantly higher in patients at stage T3–T4 than those at stage T1–T2. **d** Let-7b expression was significantly lower in patients at stage T3–T4 than those at stage T1–T2. **e** miR-205 expression was significantly higher in patients with bone metastasis than in those without. **f** Let-7b expression was significantly higher in hormone-dependent group than in hormone-resistant group: before, prostate cancer patients treated without PSA; after, prostate cancer patients treated without PSA. PSA < 4, PCa patients treated with PSA < 4 ng/ml; PSA > 4, PCa patients treated with PSA > 4 ng/ml. T1–T2, low TNM score group; T3–T4, high TNM score group. Dependent, PCa patients in the hormone-dependent group; resistant, PCa patients in the hormone-resistant group. Results are shown as means ± SD. *p < 0.05

In a series of subgroup analyses, we examined changes in miRNA levels before and after treatment within the same PCa patient group. Only miR-1825 expression was shown to change significantly following treatment, with a significant decrease observed (Fig. 3a, Fig. S1, and Table S6).

### miRNA levels in PCa patients according to PSA levels

We next examined miRNA levels in PCa patients stratified according to PSA levels regardless of any treatment: the PSA < 4 ng/ml group and PSA > 4 ng/ml group. Only the expression of let-7b differed significantly between the two groups, being significantly higher in the PSA < 4 ng/ml group than in the PSA > 4 ng/ml group (Fig. 3b, Fig. S2, and Table S7).

### miRNA levels according to tumor–node–metastasis (TNM) score

We also examined miRNA levels in PCa patients divided according to TNM stage. We found that miR-1825 expression was significantly increased in T3–T4 stage patients than T1–T2 stage patients (Fig. 3c and Table S8). By contrast, let-7b expression was significantly decreased in T3–T4 stage compared with T1–T2 stage patients (Fig. 3d, Fig. S3, and Table S8).

### miRNA levels according to bone metastasis

Bone metastasis in PCa patients was diagnosed using computed tomography (CT), magnetic resonance imaging, and emission CT. Examining miRNA levels in patients according to the presence or absence of bone metastasis showed that only miR-205 was significantly different between the two groups, being higher in patients with bone metastasis than without (Fig. 3e, Fig. S4, and Table S9).

### miRNA levels in hormone-dependent and -resistant patients

After treatment, hormone-dependent PCa patients showed normal levels of PSA (< 4 ng/ml). However, PSA levels rose again in some PCa patients who received endocrine therapy, indicating that they were hormone-resistant. We observed a significant difference in let-7b expression between hormone-dependent and hormone-resistant patients with lower level in hormone-resistant group than hormone-dependent group (Fig. 3f, Fig. S5, and Table S10).

### Receiver operating characteristic (ROC) curve analysis of the diagnostic value of miR-1825, miR-484, miR-205, miR-141, and let-7b in PCa

miR-1825 expression was shown to be able to distinguish PCa patients from healthy controls, with ROC curve analysis revealing a cut-off value of 1.368, a specificity of 91.2%, a sensitivity of 93.1%, an area under the curve of 0.957, and a 95% confidence interval of 0.916–0.997 (Fig. S6a). Similar analyses for other miRNAs revealed values of 0.590, 69.4%, 88.2%, 0.786, and 0.702–0.871, respectively, for miR-484 (Fig. S6b), 0.668, 100%, 77.78%, 0.914, and 0.860–0.967, respectively, for miR-205 (Fig. S6c), 2.459, 100%, 87.5%, 0.929, and 0.873–0.984, respectively, for miR-141 (Fig. S6d), and 0.972, 88.2%, 72.2%, 0.848, and 0.775–0.921, respectively, for let-7b (Fig. S6e).

## Discussion

At present, PSA testing is widely used in the clinic for the diagnosis of PCa, but it is not sufficient for an accurate early diagnosis and prognosis. Moreover, some treatments including BPH, glandular inflammation, drug therapy, prostate massage, and the finger test can directly affect serum PSA levels [2]. Additionally, certain types of PCa, such as small cell carcinoma, mostly show normal PSA levels [14]. PSA measurements have also been responsible for misdiagnosis and overtreatment, thereby increasing the unnecessary pain of patients and wasting medical resources. Although PCa is occasionally identified during the pathological examination of BPH specimens, we urgently require a sensitive and accurate biological marker to objectively evaluate the condition with the aim of achieving precise medical treatment.

Multiple studies have recently established an important role for miRNAs in the biological processes of prostate tumors [15]. Mitchell et al. [3] found that tumor-derived miRNAs could stably exist in blood, while Chen et al. [16] recently showed that some miRNAs could accurately distinguish PCa and BPH. Although many studies have investigated miRNAs expressed in PCa [17, 18], few have examined levels before and after treatment, with or without metastasis, or according to the degree of malignant tumor.

In the present study, we found a significantly higher level of miR-1825 expression in the serum of PCa patients than healthy controls. This differed from the results of Taha, and may reflect the analysis of different tissue types or the small sample size [5]. miR-1825 is known to target the inhibition of discoidin domain receptor (DDR)1, which participates in some epithelial-related cancers and may play a role in insulin-like growth factor-1 receptor functional cross talk in cancer progression [19, 20]. Abnormal miR-1825 levels may result in the accelerated translation and increased expression of DDR1, leading to the induction of tumorigenesis. We also found significantly lower levels of miR-1825 expression in PCa patients after treatment compared with before, while PCa patients with a higher TNM grade had significantly higher miR-1825 levels than those with a lower TNM grade. These results suggest that the miR-1825 level is positively correlated with the malignant degree of PCa tumors, which may be useful as a criterion for diagnosis and the evaluation of PCa treatment.

We also found that the expression of serum miR-484 in was significantly lower in PCa patients compared with healthy controls. Many studies have reported that the miR-484 functional target gene might be *UBR5*, which is a key regulator in cell signaling [21, 22]. This could explain the mechanism of miR-484 in the occurrence and progression of tumors. We only identified a single article about miR-484 in PCa, which identified abnormal levels in patient urine [5]. Our study provides further evidence that miR-484 has the potential to diagnose PCa.

miR-205 is mainly expressed on the basal cells of prostate tissue and is down-regulated in PCa, which might inhibit tumor growth [23–25]. We detected significantly lower miR-205 expression in PCa patients than in healthy controls, and observed a sensitivity of 77.8% for miR-205 in the diagnosis of distant tumor metastasis, and a specificity of 100%. Previous studies showed that miR-205 is not only involved in the regulation of tumor cell migration, but that its expression is negatively correlated with tumor metastasis [24]. miR-205 levels were also lower in PCa patients with bone metastases [25], which is in line with our current results. We also showed that miR-205 expression was significantly lower in patients with metastatic PCa than in control. miR-205 levels could therefore be used in the early diagnosis of PCa and the detection of bone metastases.

Tumor-related mutations often occur in the chromosomal region of human let-7b [26], which contributes to the suppression of tumor genes [27]. Previous studies showed that the let-7 family plays an important role in the recurrence and metastasis of PCa by regulating tumor stem cells [8], and in the hormone resistance of tumors [9]. Specifically, let-7b was previously reported to inhibit the growth of tumor stem cells by regulating EZH2 [28]. We observed significantly lower let-7b expression in PCa patients compared with healthy controls, and detected significantly lower levels in patients with high PSA levels, higher TNM staging, and hormone resistance. Our findings indicate that let-7b may be useful as an early diagnostic index for tumor diagnosis, the extent of tumor malignancy, and the assessment of failed hormone therapy. It could also be used to aid early diagnosis and adjust individualized treatment regimens for high-risk PCa.

Additionally, we demonstrated a significant increase in miR-141 levels in PCa patients compared with controls, which was consistent with a previous analysis of serum miRNA expression profiles in PCa patients [29]. AR is a possible target gene of miR-141-3p, which provides an effective focus for targeted PCa treatment [11, 12]. Our findings suggested that serum miR-141 may be a useful prognostic marker for PCa, although it did not appear to distinguish specific patient subgroups.

Our study identified and characterized several potential miRNA markers for PCa, including miR-1825, miR-484, miR-205, miR-141, and Let-7b. miR-205 may also be used as a biomarker for indicating bone metastasis in PCa patients, miR-1825 to indicate tumor TNM classification, evaluate treatment effects, and suggest prognosis, while let-7b levels may indicate tumor malignancy and hormone resistance status and be used to adjust individual treatments for the high-risk, early diagnosis of refractory PCa.

## Electronic supplementary material

Below is the link to the electronic supplementary material.

Supplementary material 1 (DOCX 303 KB)
