# Supplementary material for: Five microRNAs in serum as potential biomarkers for prostate cancer risk assessment and therapeutic intervention
Source: Int Urol Nephrol. 2018 Oct 15;50(12):2193–200. doi: 10.1007/s11255-018-2009-4 (PMC6267169; doi:10.1007/s11255-018-2009-4)
Supplement: Supplementary file 1 — Supplementary material 1 (DOCX 303 KB) [file 11255_2018_2009_MOESM1_ESM.docx]

**Five microRNAs in serum as potential biomarkers for prostate cancer risk assessment and therapeutic intervention**

Xiaogang Guo^1#^, Tao Han^1#^, Pingping Hu^2^, Xiaojun Guo^1^, Changming Zhu^1^, Youbao Wang^1^, Shaoyan Chang^3*^

1 Urology Department of Urology, Haici Medical Group of Qingdao, Qingdao, Shandong Province, China

2 Department of Cardiology, Jimo People’s Hospital, Qingdao City, Shandong Province, China

3 Beijing Municipal Key Laboratory of Child Development and Nutriomics, Capital Institute of Pediatrics, Beijing, China

*Corresponding author: [changsyan_2001@163.com](mailto:changsyan_2001@163.com)

**^#^**These authors contributed equally to this work.

**Supplementary results**

In addition, we have also done other stratification analysis. PCa patients were divided according to treatment: patients who were diagnosed with PCa and under no treatment were included in the untreated group, and pathologically diagnosed patients who were treated with endocrine, surgery, radiotherapy or combined therapy for > 1 month were included in the treated group. We did not find any statistical significant differences in expressions in all miRNAs examined between both groups (Table S11).

We also examined miRNA levels in PCa patients divided according to Gleason score: patients with Gleason score <=7 points were in the low-risk group, and patients with Gleason >=8points were in the high-risk group. We did not find any statistical significant differences in expressions in all miRNAs examined between both groups. (Table S12).

**Table S1** **Reverse transcriptional reaction system**

| **component** | **ultimate density** | **dosage** |
| --- | --- | --- |
| 2×Reverse transcriptant buffer | 2× | 10μl |
| Reverse transcription primer | 50nM | 1.2μl |
| Total RNA | — | 2μl |
| reverse transcriptase (200U/μl) | 2 U/μl | 0.2μl |
| Diethy pyrocarbonat water |  | To 20μl |

**Table S2 Primer sequences for real-time PCR**

| **micro RNAs** | **Forword primers** | **Reverse primers** |
| --- | --- | --- |
| hsa-miR-205 | CATACCTCCTTCATTCCACCG | TATGGTTGTTCTCGTCTCCTTCTC |
| hsa-miR-1825 | ACCTCTAAGTCCAGTGCCCTC | TATGCTTGTTCTCGTCTCTGTGTC |
| hsa-let-7b | CGTTCGTGAGGTAGTAGGTTGTG | TATGGTTGTTCTCGTCTCCTTCTC |
| hsa-miR-141 | CATCCGATTAACACTGTCTGGTAA | TATGGTTGTTCTGCTCTCTGTCTC |
| hsa-miR-484 | CAATCCTCTCAGGCTCAGTCC | TATGCTTGTTCTCGTCTCTGTGTC |
| Cel-miR-39 | GACTTCATCACCGGGTGTAAATC | TATCGTTGTTCTCCACTCCTTGAC |

**Table S3 Real-time fluorescence quantitative response system**

| **component** | **ultimate density** | **dosage** |
| --- | --- | --- |
| 2× Quantitative PCR Master Mix | 1× | 10μl |
| forward primer(20 uM) | 0.08μM | 0.08μl |
| reverse primer(20 uM) | 0.08μM | 0.08μl |
| cDNA template | — | 2μl |
| Taq DNA polymerase (2.5U/μl) | 0.05 U/μl | 0.4μl |
| Distillation-Distillation H2O |  | Add to 20μl |

**Table S4 miRNA relative expression levels between prostate cancer and control groups.**

| micro RNAs | Control（n=34） | PCa （n=72） | *p* |
| --- | --- | --- | --- |
| miR-1825 | 1.02±0.26 | 2.53±1.96 | <0.001^a^ |
| miR-484 | 0.75±0.14 | 0.53±0.32 | <0.001^a^ |
| miR-141 | 1.36±0.45 | 8.07±7.89 | <0.001^a^ |
| miR-205 | 1.10±0.37 | 0.49±0.32 | <0.001^b^ |
| Let-7b | 1.31±0.34 | 0.81±0.38 | <0.001^b^ |

a, t test; b, rank test

**Table S5 miRNA relative expression levels between PCa patients treated with the drug of PSA (< 4 ng/ml) and control groups.**

| micro RNAs | Control（n=34） | PCa with PSA<4（n=22） | *p* |
| --- | --- | --- | --- |
| miR-1825 | 1.02±0.26 | 2.39±1.72 | <0.001^a^ |
| miR-484 | 0.75±0.14 | 0.46±0.24 | <0.001^a^ |
| miR-141 | 1.36±0.45 | 7.61±8.93 | <0.001^a^ |
| miR-205 | 1.10±0.37 | 0.53±0.30 | <0.001^b^ |
| Let-7b | 1.31±0.34 | 1.01±0.39 | 0.002^b^ |

a, t test; b, rank test

**Table S6 miRNA relative expression levels in PCa patients before and after PSA treatment.**

| micro RNAs | Before treatment(n=11) | After treatment(n=11) | *P^#^* |
| --- | --- | --- | --- |
| miR-1825 | 2.60±1.05 | 1.81±0.42 | 0.03 |
| miR-484 | 0.60±0.31 | 0.43±0.21 | 0.341 |
| miR-141 | 7.59±8.76 | 6.80±9.58 | 0.491 |
| miR-205 | 0.53±0.31 | 0.45±0.23 | 0.622 |
| Let-7b | 0.98±0.40 | 0.85±0.26 | 0.309 |

#Paired t test

**Table S7 miRNA relative expression levels in PCa patients treated with different PSA concentration**

| micro RNAs | PSA treatment | | *P^#^* |
| --- | --- | --- | --- |
|  | <4ng/ml (n=22) | >4ng/ml (n=50) |  |
| miR-1825 | 2.39±1.72 | 2.61±2.07 | 0.236 |
| miR-484 | 0.46±0.24 | 0.56±0.35 | 0.299 |
| miR-141 | 7.61±8.93 | 8.32±7.44 | 0.158 |
| miR-205 | 0.53±0.30 | 0.48±0.32 | 0.533 |
| Let-7b | 1.01±0.39 | 0.71±0.33 | 0.001 |

# Rank t test

**Table S8 miRNA relative expression levels between two PCa patients’ groups with different TNM score.**

| micro RNAs | TNM score | | *P^#^* |
| --- | --- | --- | --- |
|  | T1-T2 (n=39) | T3-T4 (n=33) |  |
| miR-1825 | 2.39±1.80 | 2.70±2.15 | 0.037 |
| miR-484 | 0.47±0.27 | 0.61±0.36 | 0.109 |
| miR-141 | 7.34±7.36 | 8.93±8.51 | 0.317 |
| miR-205 | 0.54±0.34 | 0.43±0.29 | 0.093 |
| Let-7b | 0.88±0.38 | 0.74±0.39 | 0.033 |

# Rank t test

**Table S9 miiRNA relative expression levels between two PCa patients’ groups according to bone metastasis.**

| micro RNAs | Bone metastasis | | *P^#^* |
| --- | --- | --- | --- |
|  | yes (n=34) | no (n=38) |  |
| miR-1825 | 2.14±1.05 | 2.89±2.47 | 0.068 |
| miR-484 | 0.52±0.27 | 0.55±0.36 | 0.933 |
| miR-141 | 7.45±8.88 | 8.63±6.96 | 0.084 |
| miR-205 | 0.55±0.26 | 0.44±0.36 | 0.012 |
| Let-7b | 0.83±0.36 | 0.80±0.41 | 0.223 |

# Rank t test

**Table S10 miRNA relative expression levels between hormone-dependent and hormone-resistant PCa patients.**

| micro RNAs | Hormone reactivity | | *P^#^* |
| --- | --- | --- | --- |
|  | dependence (n=23) | resistance (n=25) |  |
| miR-1825 | 2.35±1.69 | 2.60±2.41 | 0.369 |
| miR-484 | 0.46±0.24 | 0.58±0.42 | 0.403 |
| miR-141 | 7.33±8.83 | 7.51±6.76 | 0.322 |
| miR-205 | 0.51±0.31 | 0.47±0.26 | 0.757 |
| Let-7b | 1.03±0.40 | 0.64±0.27 | <0.001 |

# Rank t test

**Table S11 miRNA relative expression levels between two PCa patients’ groups according to PSA treatment.**

| micro RNAs | PSA treatment | | *P^#^* |
| --- | --- | --- | --- |
|  | yes (n=48) | no (n=24) |  |
| miR-1825 | 2.48±2.08 | 2.68±1.77 | 0.248 |
| miR-484 | 0.53±0.34 | 0.55±0.28 | 0.585 |
| miR-141 | 0.49±0.29 | 0.50±0.40 | 0.844 |
| miR-205 | 7.42±7.73 | 9.59±8.31 | 0.073 |
| Let-7b | 0.83±0.39 | 0.78±0.39 | 0.641 |

# Rank t test

**Table S12 miRNA relative expression levels between two PCa patients’ groups with low risk (6-7) and high risk (8-10) according to Gleason score classification.**

| micro RNAs | Gleason score | | *P^#^* |
| --- | --- | --- | --- |
|  | 6-7 (n=29) | 8-10 (n=43) |  |
| miR-1825 | 2.25±1.08 | 2.73±2.37 | 0.77 |
| miR-484 | 0.52±0.27 | 0.54±0.35 | 0.931 |
| miR-141 | 7.11±7.31 | 8.71±8.28 | 0.235 |
| miR-205 | 0.53±0.34 | 0.46±0.31 | 0.312 |
| Let-7b | 0.80±0.37 | 0.82±0.40 | 0.904 |

# Rank t test

**

**

**Fig.S1** **miRNA relative expression levels in PCa patients before and after PSA treatment.** a-d) There were no significantly difference in the relative expression of miR-484, miR-205, miR-141, Let-7b between before and after PSA treatment. before, prostate cancer patients treated without PSA; after, prostate cancer patients treated without PSA. Results were shown as ‾x±SD. **p*<0.05, ***p*<0.01





**Fig.S2** **miRNA relative expression levels in PCa patients treated with different PSA concentration.** a-d) There were no significantly difference in the relative expression of miR-1825, miR-484, miR-205, miR-141 between PCa patients treated with different PSA concentration**.** PSA<4, PCa patients treated with PSA<4 ng/ml; PSA>4, PCa patients treated with PSA>4 ng/ml. Results were shown as ‾x±SD.





**Fig.S3** **miRNA relative expression levels between two PCa patients’ groups with different TNM score.** a-c) There were no significantly difference in the relative expression of, miR-484, miR-205, miR-141 between T1-T2 and T3-T4**.** T1-T2, low TNM score group; T3-T4, high TNM score group. Results were shown as ‾x±SD.





**Fig.S4 miRNA relative expression levels between two PCa patients’ groups according to bone metastasis.** a-d) There were no significantly difference in the relative expression of miR-1825, miR-484, miR-141, Let-7b between bone metastasis and non-bone metastasis**.** Results were shown as ‾x±SD.

**

**

**Fig.S5 miRNA relative expression levels between hormone-dependent and hormone-resistant PCa patients.** a-d) There were no significantly difference in the relative expression of miR-1825, miR-484, miR-205, miR-141 between Dependent and Resistant**.** Dependent, PCa patients with hormone-dependent group; Resistant, PCa patients with hormone-resistant group. Results were shown as ‾x±SD.





**Fig.S6** **ROC of five miRNAs relative expression levels between prostate cancer and control groups**. ROC , receiver operating characteristic curve.
